# Extracting the Temperature Dependence of Both Nanowire Resistivity and Junction Resistance from Electrical Measurements on Printed Silver Nanowire Networks

**DOI:** 10.1021/acsaelm.4c01965

**Published:** 2025-01-09

**Authors:** Emmet Coleman, Adam Kelly, Cian Gabbett, Luke Doolan, Shixin Liu, Neelam Yadav, Jagdish K. Vij, Jonathan N. Coleman

**Affiliations:** † School of Physics, CRANN & AMBER Research Centres, Trinity College Dublin, Dublin D02 PN40, Ireland; ‡ Department of Electronic & Electrical Engineering, Trinity College Dublin, Dublin D02 PN40, Ireland

**Keywords:** nanowires, nanotubes, solution-deposited, conductivity, interparticle

## Abstract

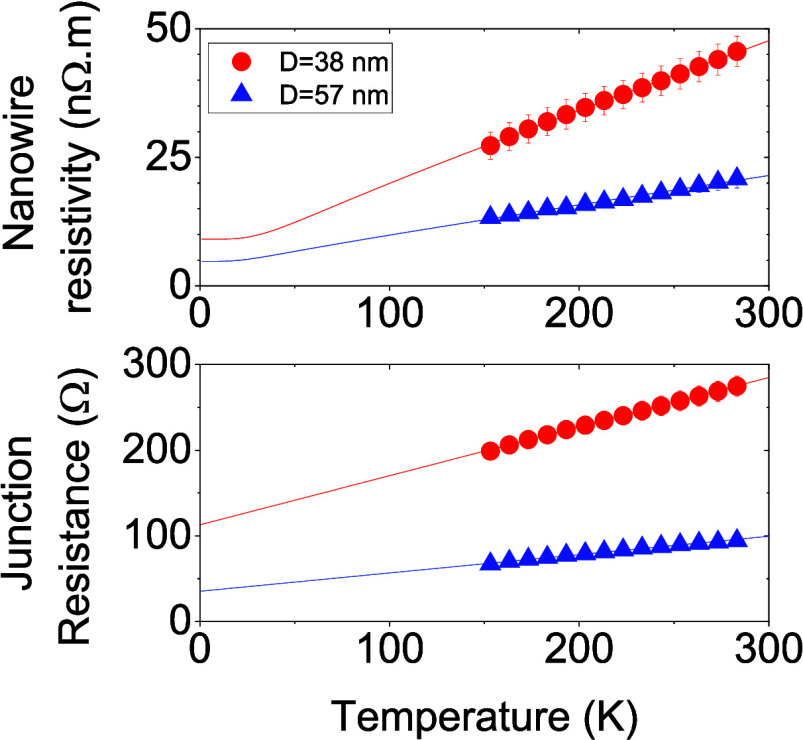

Printed networks of nanoparticles (e.g., nanodots, nanowires, nanosheets) are important for a range of electronic, sensing and energy storage applications. Characterizing the temperature dependence of both the nanoparticle resistivity (ρ_NW_) and interparticle junction resistance (*R*_J_) in such networks is crucial for understanding the conduction mechanism and so for optimizing network properties. However, it is challenging to extract both ρ_NW_ and *R*_J_ from standard electrical measurements. Here, using silver nanowires (AgNWs) as a model system, we describe a broadly applicable method to extract both parameters from resistivity measurements on nanowire networks. We achieve this by combining a simple theoretical model with temperature-dependent resistivity measurements on sets of networks fabricated from nanowires of different lengths. As expected, our results demonstrate that *R*_J_ is the predominant bottleneck for charge transport within these networks, with *R*_NW_/*R*_J_ in the range 0.03–0.7. We demonstrate that the temperature dependence of ρ_NW_ exhibits characteristic Bloch–Grüneisen behavior, yielding a Debye temperature between 133–181 K, which aligns with reported values for individual nanowires. Likewise, our findings for residual resistivity and electron–phonon coupling constants closely match published values measured on individual nanowires. The junction resistance also follows Bloch–Grüneisen behavior with similar parameters, indicating the junctions consist of metallic silver. These findings confirm the validity of our method and provide a deeper insight into the conduction mechanisms in AgNW networks. They also pave the way toward simultaneous measurement of ρ_NW_ and *R*_J_ in other important systems, notably carbon nanotube networks.

## Introduction

The ability to solution-deposit electronic devices, components and circuitry has led to the rapidly expanding field of printed electronics (PE). Although printed electronics devices tend to have reduced performance compared to traditional electronics, they display considerable advantages in terms of flexibility, area-scalability, and cost effectiveness.^1,2^ Due to their combination of solution-processability and their excellent electrical performance, a range of nanoparticles - including 0D nanodots, one-dimensional (1D) nanowires/nanotubes and two-dimensional (2D) nanosheets - have been extensively explored in a range of PE applications.^1,3−5^ Printed materials and additive manufacturing has developed into an area of great interest^6−8^ and in particular printed nanoparticle networks have been utilized in various applications including highly conductive transparent electrodes^9^ and photodetectors.^10^ Alternatively, metallic nanowire networks have shown promise as flexible transparent electrodes,^11−14^ heating elements,^15,16^ wearable electronics,^17^ battery and capacitor electrodes,^5,18,19^ sensors,^20^ and electromagnetic interference (EMI) shields^21−23^ while printed carbon nanotube networks have shown applications as transistors,^24^ light-emitting diodes (LEDs),^25^ and photodetectors.^26^ More recently, solution-deposited networks of 2D materials such as graphene and MoS_2_ have been examined for use in a wide range of fields from (opto)electronics to energy storage.^27−31^ Many of these applications require the maximization of the conductivity or mobility of the nanoparticle networks (nanonets).

It is important to study the details of charge transport in such networks, both to further our understanding of the physics of the conduction mechanisms and to facilitate the maximization of the electrical performance of such networks. However, standard methods for studying charge transport mechanisms, such as the measurement of conductivity and/or mobility as a function of temperature, do not give a complete picture of electrical conduction in these systems. This is because charge carriers traversing a nanonet must alternate between two modes of conduction; intra- and interparticle charge transport.^32−34^ Clearly, carriers must spend some time traveling through the nanoparticles (intraparticle transport). This might be via bandlike transport in ordered materials or via hopping in disordered or defective ones. However, after traveling through a nanoparticle, it will always be necessary for the carrier to transit to the next nanoparticle, i.e., to cross a particle–particle junction. We refer to this as interparticle transport and, in most cases,^27,34^ it will be via hopping or tunneling (but not always, see below). Movement of carriers over long distances in nanonets will always involve alteration between intra- and interparticle transport. This means the basic unit of resistance associated with a nanonet is *R*_J_ + *R*_N_ where *R*_N_ is the resistance of the (average) nanoparticle and *R*_J_ is the resistance of the (average) junction.^32^ Thus, because temperature-dependent network resistivity measurements probe *R*_J_ + *R*_N_, it is difficult to separate the effects of intra- and interparticle charge transport.

Usually, nanonets display network conductivity and mobility that tend to be at least an order of magnitude below that of the component nanoparticles.^27,34^ It is usually assumed that this is because transport across junctions is rate-limiting i.e., *R*_J_ ≫ *R*_N_. This simplifies analysis of the temperature-dependence of network conductivity or mobility by allowing researchers to make the approximation that any mechanistic information contained in the data is associated with interparticle transport. While this is probably true for most nanonets today, it will not be the case as networks improve and junction resistances are reduced to the point where *R*_J_*∼ R*_N_.

To properly study the charge transport mechanism in nanonets, it is necessary to separately characterize both intraparticle and interparticle charge transport mechanisms. Put simply, both *R*_J_ and *R*_N_ must be measured together as a function of temperature (or magnetic field or gate voltage etc.). The first steps in this direction have been demonstrated recently. We have recently described a method^32^ based on impedance spectroscopy to simultaneously measure *R*_J_ and *R*_N_ as a function of temperature in networks of semiconducting MoS_2_ nanosheets.

However, this impedance-based method is probably not applicable to networks of metallic or semimetallic nanoparticles. This is because such systems are expected to have relatively low junction resistances,^32^ shifting the features of interest within the impedance spectrum out of the measurable frequency range (see Supporting Information for further detail). Thus, an alternative method is required to simultaneously interrogate both intra- and interparticle transport mechanisms in networks of conducting nanoparticles.

Here, we will demonstrate a simple method to measure the temperature dependence of *R*_N_ and *R***_*J*_** in conducting nanonets which cannot be interrogated using standard impedance spectroscopy. To do this, we measure the resistivity of conducting nanoparticle networks at various temperatures for various nanoparticle sizes. We then use a simple model^32^ to extract *R*_N_ and *R*_J_ from the resistivity versus particle size data, at each temperature. We demonstrate this method using spray coated networks of silver nanowires (AgNWs) as a model system. AgNW networks have been heavily studied due to their excellent performance in various applications in the field of wearable technologies.^11−13,35−37^ However, we use them here because the electrical properties of individual silver nanowires are well-defined and well-known. That is, their resistance is related to their resistivity via their dimensions in a simple way, while the resistivity of individual nanowires is reasonably close to the value of bulk silver in most cases.^38^ In addition, their resistivity is not affected by environmental effects such as inadvertent doping in the way it is for other nanoparticles.^39^ This means that one can easily benchmark the values of *R*_*N*_ extracted from our model with the literature in a way that would not be possible with other nanomaterials.

## Results and Discussion

### Length Control and Measurement of Nanowire Length

**Figure 1:**
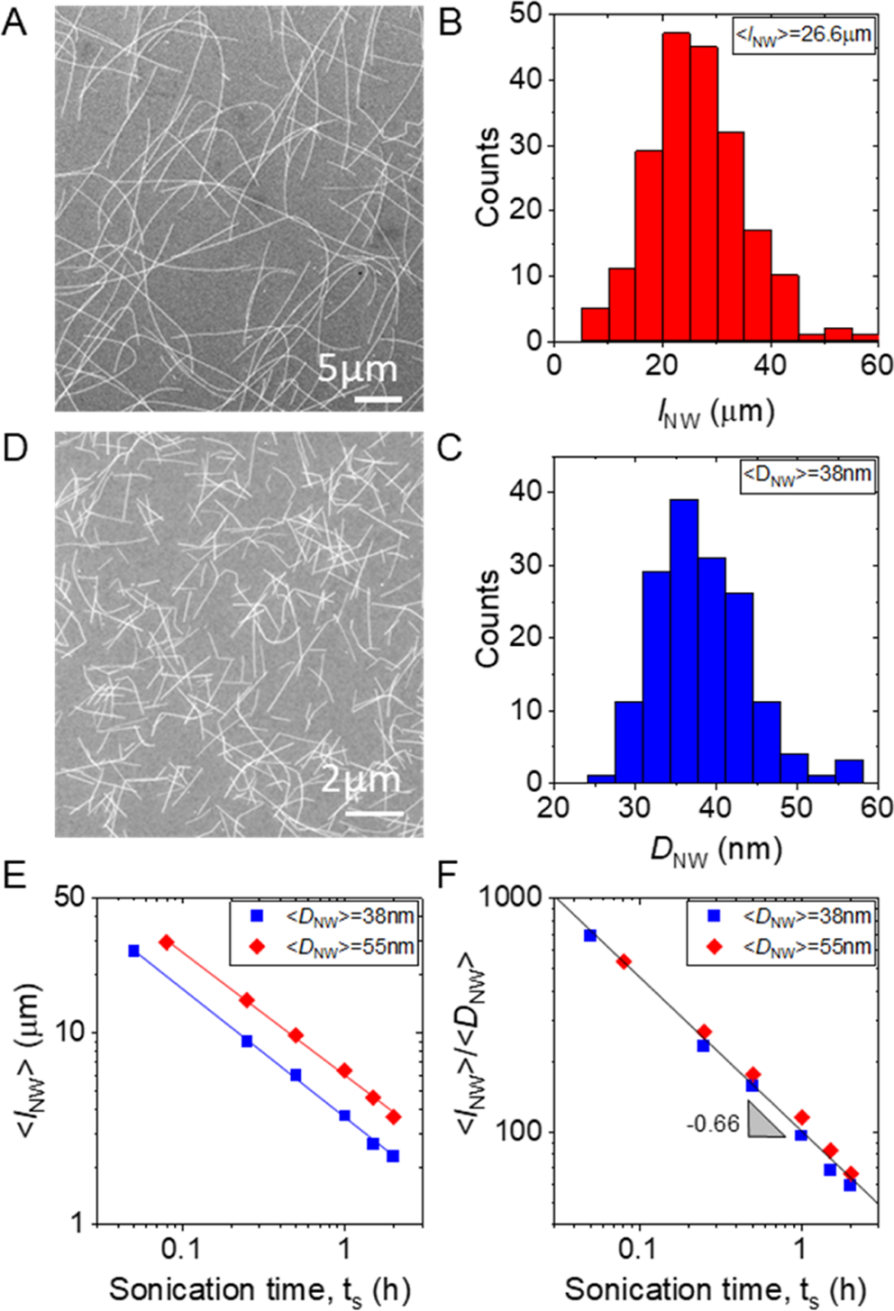
Material characterization. (A) SEM image of as-purchased AgNWs (*D*_NW_ = 38 nm) deposited on Si/SiO_2_. (B–C) Example histograms showing the length (C) and diameter (D) distributions for the nanowires shown in A. Note: Full AgNW size analysis can be found in Supporting Information XLXS. (D) SEM image of AgNWs (*D*_NW_ = 38 nm) deposited on Si/SiO_2_ after sonication for 2 h. (E) Mean length of AgNWs within each size-selected fraction as a function of sonication time. Data is shown for both *D*_NW_ = 38 nm and *D*_NW_ = 57 nm nanowires. (F) Plot of ⟨*l*_NW_⟩/⟨*D*_NW_⟩ as a function of sonication time showing data for all fractions over both nanowire diameters falls on a master curve.

A key aspect of this work will be to measure the dependence of network resistivity on nanowire length. To achieve this, batches of nanowires of two different diameters were purchased from Novarials Corporation. Scanning electron microscope (SEM) images of AgNWs deposited on substrates showed the nanowires to be long and uniform as shown in Figure 1A. Image analysis allowed the measurement of both the length and diameter of the nanowires, with both parameters showing reasonably narrow distribution as shown in Figure 1B,C (see Supporting Information (XLXS) for all statistical data). The mean diameters, *D*_NW_, of the two nanowire batches were 38 ± 1 and 57 ± 1 nm, while the corresponding mean nanowire lengths, *l*_NW_, were found to be 26.6 ± 1 and 29.3 ± 1 μm respectively (errors are standard errors of the mean).

The AgNWs were size-selected by sonicating the dispersions for various periods of time, a process known as sonication-induced scission.^16,40,41^ SEM images such as that shown in Figure 1D confirmed that this process results in shortening of the nanowires. This produced a range of fractions, each with a different mean nanowire length. SEM measurements (Figure 1E) showed the length to decrease with sonication time, from 26.6 to 2.3 μm for the 38 nm diameter AgNWs and 29.3 to 3.6 μm for the 57 nm diameter AgNWs. This falloff in length with sonication time followed a power law, consistent with previously observed behavior.^16,40,41^ The data in Figure 1E is replotted in Figure 1F as the nanowire aspect ratio, *l*_NW_/*D*_NW_, plotted versus sonication time, *t*. This graph clearly demonstrates that the sonication data for both AgNW diameters converge onto a master curve consistent with *l*_NW_ ∝ *D*_NW_ × *t*^*n*^, where *n* = −0.66. This exponent compares well to values of *n* = −0.5 obtained for single-walled carbon nanotubes (SWCNTs)^42^ and graphene^43^ but differs somewhat from a previous report of *n* = −0.34 for AgNWs.^44^

### Network Characterization

**Figure 2:**
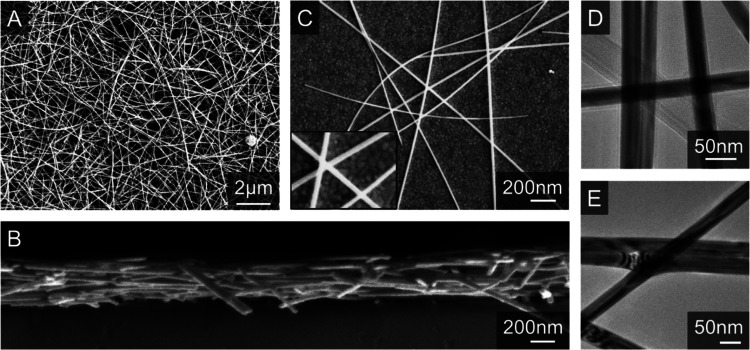
Network characterization. (A) SEM image of the top surface of stock length AgNW network with *D*_NW_ = 38 nm nanowires. (B) Typical cross-sectional SEM image of a AgNW network. (C) High Magnification SEM image of typical junctions present within a AgNW network. (D) Transmission electron microscopy (TEM) image of an unannealed AgNW–AgNW junction. (E) TEM image of a completely annealed AgNW–AgNW junction showing sintering at the junction.

Nanowire networks were produced by spray-coating. Typically, networks were sprayed with an average thickness of ∼500 nm; thick enough to avoid any percolative effects (i.e., thickness-dependent conductivity which occurs for network thicknesses below ∼100 nm for AgNWs).^45^ Spray coating produced disordered networks of randomly oriented nanowires as shown in Figure 2A,B. Such networks are also known to be highly porous with porosities of 80–90% typical.^46^

The internanowire junctions can be seen more clearly by visualizing a sparse network using SEM (Figure 2C) or TEM (Figure 2D,E). These images show the junctions as consisting of nanowires in close contact. However, it is known that such as-produced nanowire junctions contain a layer of stabilizing poly(vinylpyrrolidone) (PVP) polymer, which acts as an insulating barrier between the AgNWs. This significantly hinders electron transport in as-produced AgNW networks, causing them to have a large network resistance. Thermal annealing has been shown to drastically reduce the network resistance by allowing the PVP layer to migrate and sintering the nanowires at the junctions.^47−49^ TEM images of individual junctions in AgNW networks after thermal annealing reveal dramatic changes in the junction morphology. In Figure 2E, post anneal, there is a clear welding at the junctions, suggesting displacement of the PVP layer. Overall, the TEM images show that the annealing process enhances the intimacy of the contact between the AgNWs at the junctions, helping to drastically reduce junction resistance and improve internanowire transport. It also implies that the junctions themselves consist of metallic silver after annealing.

In order to quantify the morphology of these networks, we performed 3D imaging via nanotomography. As described in ref (46), 3D imaging of nanonetworks can be achieved using a combination of repeated focused ion beam milling and scanning electron microscopy (FIB-SEM). After the appropriate image segmentation, this procedure yields a large set of 2D images where each pixel is labeled via its material composition. These images can then be combined to generate a 3D image where each voxel represents (in our case) either free space (part of a pore) or silver (part of a nanowire). Typically, such 3D images have resolution of 5 nm in the image plane and 15 nm out of plane, i.e., the voxel size is 5 × 5 × 15 nm^3^.

**Figure 3:**
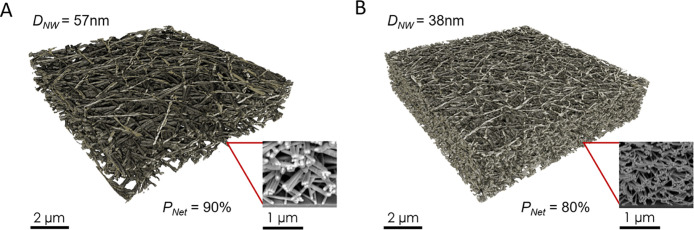
Network 3D imaging. FIB-SEM tomography images of sections of networks spray-cast from silver nanowires with diameters of *D*_NW_ = 57 nm (A) and *D*_NW_ = 38 nm (B). The insets at the bottom right of each panel show small sections of individual cross-sectional SEMs.

Examples of 3D images collected for the nanowire networks under study here are shown in Figure 3. In principle, such images contain all structural and morphological information relating to the network which is available at the resolution of the image. This includes parameters relating to nanowire orientation as well as pore size and shape etc. However, for our purposes, the most important available information relates to the network porosity (*P*_net_) because of its effect on the network resistivity (see below). Using the methods described in ref (46) we have calculated mean network porosities of *P*_net_ = 85 ± 5% for networks of AgNWs with *D*_NW_ = 38 nm and *P*_net_ = 89 ± 3% for networks of AgNWs with *D*_NW_ = 57 nm.

### Length Dependence

We recently demonstrated^32^ that the resistivity, ρ_net_, of a network of 1D nanowires (or nanotubes) is related to the resistivity of the nanowires themselves, ρ_NW_, by
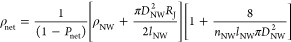
1where *P*_net_, *D*_NW_, *l*_NW_ and *n*_NW_ are mean values of network porosity, nanowire diameter, nanowire length and nanowire carrier concentration, respectively. In the case of conducting nanomaterials such as AgNWs, *n*_NW_ is very large, meaning the final term of this equation becomes negligible:
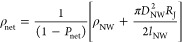
2This equation predicts that measuring the network resistivity (ρ_net_) as a function of AgNW length (*l*_NW_) will allow the extraction *R*_NW_ and *R*_J_ by fitting, once *P*_net_ and *D*_NW_ are known. The introduction of this equation serves as a foundational framework for our analyses, providing a quantitative basis for the determination of the role of junctions in AgNW networks.

**Figure 4:**
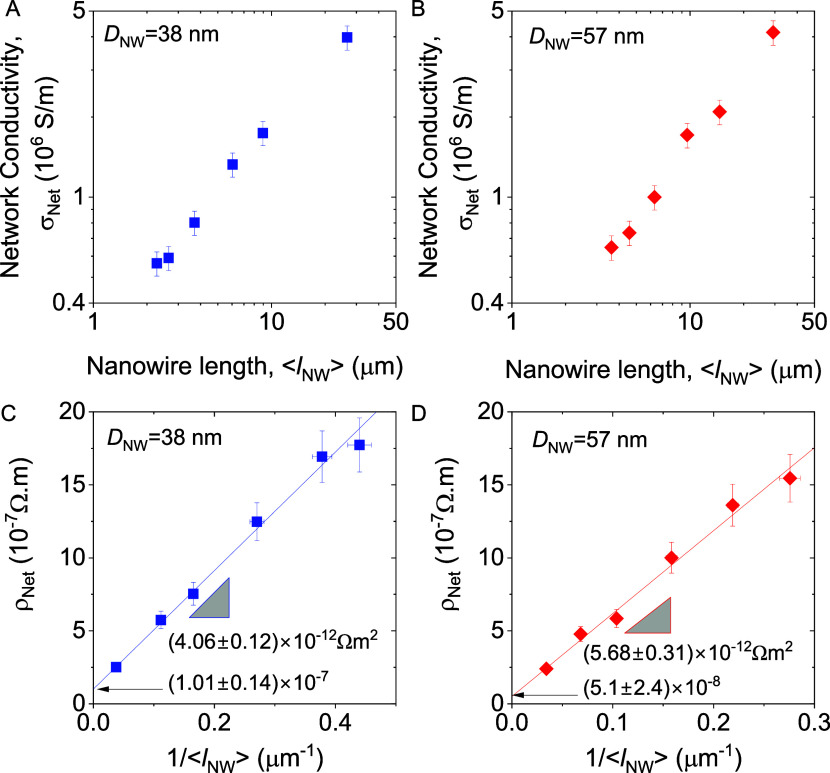
Dependence of network conductivity on AgNW length. (A, B) Conductivity of spray-cast AgNW networks, σ_net_, versus mean nanowire length, *l*_NW_, for AgNWs of diameter, *D*_NW_, 38 and 57 nm, respectively. (C, D) Resistivity of spray-cast AgNW networks, ρ_net_, versus the inverse of the mean nanowire length for AgNWs of diameter 38 and 57 nm, respectively. The line is a fit to eq 2 which allows for the extraction of ρ_NW_ and *R*_J_ from the intercept and slope, respectively.

To investigate the effect of nanowire length on the electrical properties of the network, we used the length-selected nanowire dispersions produced above to fabricate a set of networks with various nanowire lengths. We then measured the network conductivity, σ_net_, using 4-probe techniques. Figure 4A,B shows σ_net_, measured for both nanowire diameters, as a function of the mean length of nanowires making up the network, *l*_*NW*_. These graphs both appear to scale roughly linearly with AgNW length as previously observed by Sorel et al.^44^ It is also worth noting that our most conductive networks display conductivities approaching 5 × 10^6^ S/m, competitive with the best metallic nanowire networks^13,48,49^ and to our knowledge surpassed only by networks of silver nanosheets.^50^

However, we note that eq 2 predicts the network resistivity, ρ_net_, to scale linearly with the inverse of nanowire length. Plotting the data as ρ_net_ versus 1/*l*_NW_ in Figure 4C,D shows straight lines for both nanowire diameters. Linear fitting allows us to extract values for ρ_NW_ and *R*_J_ for each nanowire type. We obtained values of ρ_NW_ = 1.5 ± 0.7 × 10^–8^ Ω m and ρ_NW_ = 0.6 ± 0.3 × 10^–8^ Ω m for the 38 and 57 nm AgNW networks, respectively. These values are broadly consistent with the resistivity of bulk silver (ρ_Ag_ = 1.6 × 10^–8^ Ω m), supporting the validity of our approach. The higher resistivity observed for the 38 nm nanowires is consistent with expected size-dependent surface scattering effects in smaller diameter nanowires.^51^

In addition, the junction resistances determined from our study are *R*_J_ = 265 ± 40 and 124 ± 22 Ω for the 38 and 57 nm AgNW networks, respectively. The junction resistance increases for smaller diameter nanowires due to the reduced contact area at the junction interface. These data are consistent with a wide range of values of *R*_J_ reported by Forro et al.^38^ for silver and copper nanowire networks which mostly cluster in the range 50 Ω < *R*_*J*_ < 2 kΩ. This range is also consistent with a number of experimental studies which have reported *R*_J_ values from 11 Ω to 2 kΩ.^52−56^ The wide range of reported *R*_J_ values may be attributed to the wide range of possible junction morphologies. Such a broad range of junction types is expected due to variations of nanowire diameter and, more importantly, annealing conditions.

We can use the known dimensions of our AgNWs, combined with the nanowire resistivities to estimate the nanowire resistances, *R*_NW_. To do this, we assume that carriers travel, on average, through half the length of each nanowire,^32^ leading to *R*_NW_ = ρ_NW_ (*l*_NW_/2)/(π*D*_NW_^2^/4). This allowed us to calculate *R*_NW_/*R*_J_, a parameter that determines how junction limited each network is, for each of our nanowire fraction. We found values of *R*_NW_/*R*_J_ ranging from 0.06 to 0.7 for the *D*_NW_ = 38 nm AgNW networks and from 0.03 to 0.26 for the *D*_NW_ = 57 nm AgNW networks. We note that all networks studied here have *R*_NW_/*R*_J_ < 1, showing them to be predominantly junction limited.

### Temperature Dependence of AgNW Network Resistivity

Measurement of the temperature dependence of a material’s conductivity, resistivity or mobility is a powerful tool for identifying the dominant conductive mechanisms present in the sample. A number of studies have reported the temperature dependence of the conductivity or resistivity of various solution processed networks, including those comprising AgNWs,^57^ carbon nanotubes,^58^ graphene,^59,60^ and semiconducting 2D materials such as MoS_2_.^59,61^ In each case the results reflect the temperature dependence of the rate-limiting step in the conduction process, most likely charge transport across junctions. The ability to differentiate the contributions of intraparticle transport (e.g., through the nanowires) from interparticle transport (e.g., across junctions) to the network’s overall conductivity would allow us to individually examine both intraparticle and interparticle conduction mechanisms at the same time. By separating the contributions of these two components, we can gain a more comprehensive understanding of the conduction mechanisms operating within the network.

**Figure 5:**
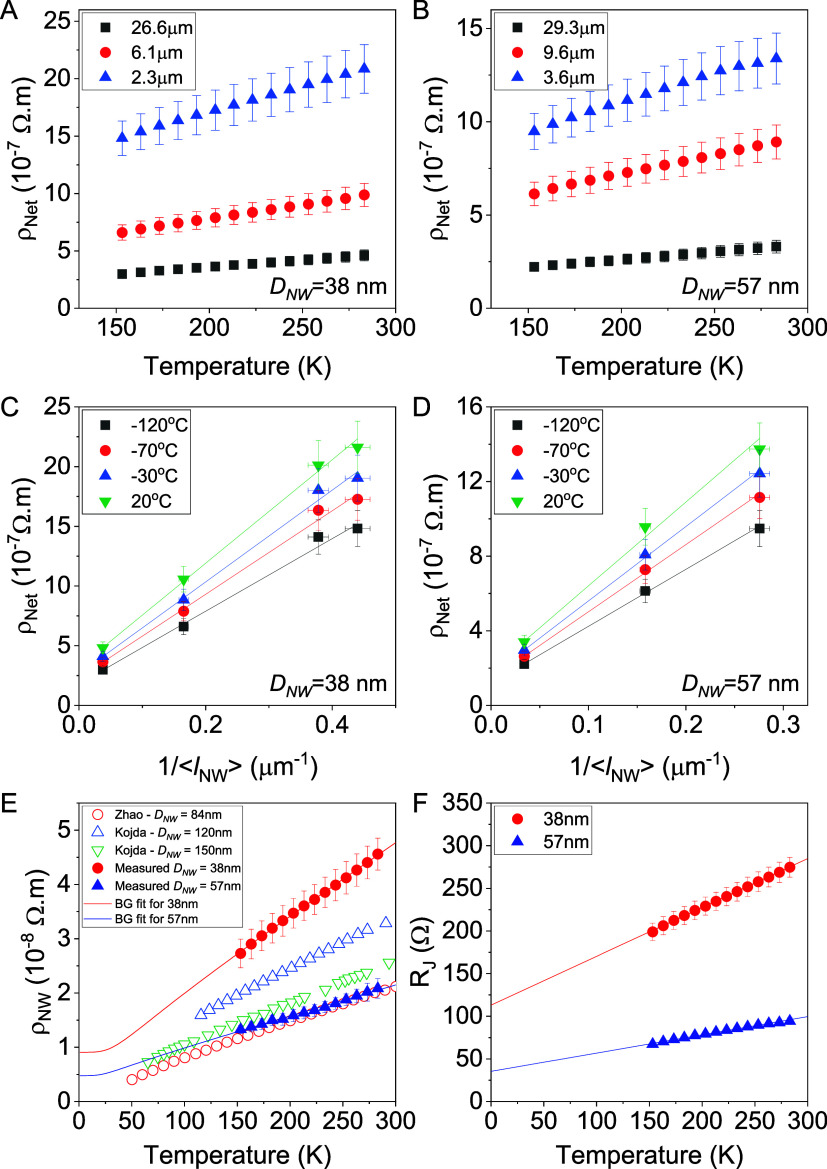
Temperature dependence of AgNW networks. (A, B) Examples of network resistivity, ρ_net_, vs temperature for networks with various AgNW lengths for nanowires with *D*_NW_ = 38 nm (A) and *D*_NW_ = 57 nm (B). (C, D) Examples of network resistivity, ρ_net_, measured at various temperatures plotted versus the inverse of the nanowire length, ⟨*l*_NW_⟩^–1^, for AgNWs of diameter 38 nm (C) and 57 nm (D). The lines are fits to eq 1. (E) The extracted nanowire resistivity plotted versus temperature for our AgNW networks compared to temperature-dependent resistivity of individual silver nanowires reported in the literature.^62,63^ The lines are a fit to eq 3 (F) The extracted junction resistance versus temperature for our AgNW networks. The lines are fits to eq 6.

Here, we achieve this by measuring the temperature dependence of the network resistivity for networks comprising nanowires of different lengths. We note that for technical reasons we used two-probe rather than 4-probe measurements, meaning our resistivity values are slightly overestimated. However, this does not affect the general validity of the method. Figure 5A,B shows the temperature dependence of the resistivity of AgNW networks for three different nanowire lengths each for AgNWs with *D*_NW_ = 38 and 57 nm, respectively. The data shows that the network resistivity increases with increasing temperature, as is characteristic of metallic materials. Such data has been reported before by various authors.^57,59^

Our novel contribution is to replot this data, not as ρ_net_ versus *T* for various nanowire lengths, but to plot ρ_net_ versus 1/*l*_NW_ for various temperatures (Figure 5C,D). This allows us to fit the data for each temperature using eq 2, as we did above. In this way, values of *R*_J_ and ρ_NW_ can be extracted for each temperature (Figure 5E,F). This allows us to examine the temperature dependence of *R*_J_ and ρ_NW_ individually, which enables the separate examination of intrananowire and internanowire conduction mechanisms.

Figure 5E shows the extracted ρ_NW_ vs temperature data, as well as literature data for ρ_*NW*_ vs temperature measured on individual AgNWs for comparison. We find our ρ_NW_ data (extracted from network measurements) to be consistent to the published data measured on individual nanowires.^62,63^

The temperature dependence of individual AgNWs has been extensively studied. It is well understood that, within certain limitations,^62^ it can be described by a combination of the Bloch–Grüneisen (BG) formula^62−64^
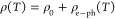
3
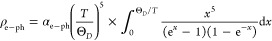
4where ρ_0_ is the residual resistivity due to defect scattering, ρ_e–ph_ is the resistivity arising from electron–phonon (e–ph) interactions, α_e–ph_ is a constant characterizing e–ph coupling and Θ_*D*_ is the Debye temperature. Equation 4 assumes that the dominant source of temperature dependence is the scattering of electrons by phonons and predicts that the resistivity of a material increases with increasing temperature. This is because the increased thermal energy allows the phonons to scatter electrons more effectively, which reduces the mean free path of electrons.

Our ρ_NW_*(T)* data shows a good fit to the BG equation over our temperature range (Figure 5E), which confirms that the primary mode of scattering in our AgNWs is due to phonons. The Debye temperature for AgNWs with *D*_NW_ = 38 and 57 nm were found to be 181 and 133 K, respectively, which aligns well with the reported range of 128–215 K for AgNWs^62−64^ and demonstrating the expected higher Debye temperature for smaller nanowires.^63,64^ The extracted residual resistivities were 8.9 × 10^–9^ Ω m for the 38 nm nanowires and 4.8 × 10^–9^ Ω m for the 57 nm nanowires, both consistent with the typical reported range of 1.7 × 10^–9^ to 2.0 × 10^–8^ Ω m for AgNWs^62−64^ and consistent with previously reported nanowire diameter scaling.^63^ Additionally, the electron–phonon coupling constants were determined to be 9.6 and 3.0 × 10^–8^ Ω m for the *D*_NW_ = 38 and 57 nm AgNWs, respectively, which also scale with *D*_NW_ as expected^63^ and fall within the reported range of 3.5 to 9.9 × 10^–8^ Ω m.^62−64^ This clearly shows that the ρ_NW_(*T*) data extracted using our method is consistent both with other reported experimental data and with the standard theoretical framework (the BG equation) used to analyze such data.

A plot of *R*_J_ vs temperature is shown in Figure 5F. The data are quite linear in *T*, consistent with metallic behavior at temperatures above the Debye temperature. We interpret this as meaning the junctions consist of silver welds as previously reported for annealed networks.^65,66^ However, this linearity, coupled with the limited temperature range, makes it impossible to apply the BG fit accurately. Nevertheless, we can roughly analyze the data by taking the high temperature approximation to eq 4 (expanding the exponentials to first order and integrating) which yields
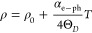
5We then assume that the junction resistance can be related to the resistivity of the material making up the junctions via *R*_J_ = ρ/*b*. Here *b* is a parameter with dimensions of length which describes the geometric properties of the junction. We can then write
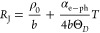
6We estimate b by comparing the values for junction resistance and nanowire resistivity at room temperature yielding *b* = 1.65 and 2.18 × 10^–10^ m for the 38 and 57 nm diameter nanowires, respectively.

We can use eq 6 to fit the data in Figure 5F. From the intercepts we find the residual resistivities to be 1.9 × 10^–8^ Ω m for the 38 nm nanowires and 7.7 × 10^–9^ Ω m for the 57 nm nanowires, both consistent with the values extracted from the ρ_NW_ data. From the slopes, we find the ratio of the electron–phonon coupling and the Debye temperature, α_e–ph_/Θ_*D*_, obtaining values of 3.8 and 1.9 × 10^–10^ ΩmK^–1^, for the 38 and 57 nm diameter nanowires respectively, close to the values of 5.3 and 2.3 × 10^–10^ ΩmK^–1^ found from the ρ_NW_ data. This result shows that the temperature dependence of our junction resistance values is fully consistent with the expected behavior of metallic silver junctions. We reiterate that such metallic junctions are expected to occur in annealed networks of silver nanowires such as those used here.^65^ These results fully support the effectiveness of our method to extract both nanowire and junction resistance from length-dependent network resistivity measurements.

## Conclusions

This study provides a broadly applicable method for the extraction of the temperature-dependent nanoparticle resistivity and junction resistance in printed nanomaterial networks, using AgNWs as a model system. By employing a nanowire length-dependent study, we determined that the junction resistance is the primary bottleneck in the charge transport within these AgNW networks, with networks exhibiting *R*_NW_/*R*_J_ < 1. Repeating these measurements at various temperatures allows us to extract both nanowire resistivity as well as junction resistance as a function of temperature. Both parameters followed Bloch–Grüneisen behavior, consistent with metallic conduction in both nanowires and junctions.

Our work emphasizes the importance of understanding the role of charge transport in both junctions and nanoparticles for the optimization of nanomaterial networks. We establish a framework for the in-depth analysis of the charge transport in nanomaterial networks which enables future work to focus on exploring ways to further optimize ρ_NP_ and *R*_J_ for improved performance in practical applications. We anticipate that this method will be used for analysis of other technologically relevant systems such as networks of semiconducting or metallic carbon nanotubes.

## Experimental Section

### AgNW Network Production

Silver nanowire stock dispersions (A40 40 nm × 35 μm in 2-propanol and A60 60 nm × 45 μm in 2-propanol) were sourced from Novarials Corporation. The AgNWs were size-selected using sonication-induced scission in an ultrasonic bath. In each case a stock AgNW dispersion was sonicated for a fixed duration at a concentration of 1 mg mL^–1^ in IPA. Sonication times of 0.05, 0.25, 0.5, 1, 1.5, and 2 h were used to produce the size-selected AgNW inks. Spray coating was carried out at a AgNW concentration of 0.5 mg mL^–1^ using a Harder and Steenbeck Infinity CRplus Airbrush attached to a computer-controlled Janome JR2300N mobile gantry. All deposited traces were defined using stainless steel shadow masks producing a serpentine pattern with a channel length of 97 mm and channel width of 500 μm. Fused silica substrates were used due to their low roughness. An N2 back pressure of 45 psi, nozzle diameter of 400 μm and stand-off distance of 100 mm between the nozzle and substrate were used.^67^ The networks were then annealed at a temperature of 135 °C to reduce *R*_J_ by sintering the junctions.

### Microscopy (SEM)

Scanning electron microscopy (SEM) of the printed nanosheet networks was performed using a Carl ZEISS Ultra Plus SEM. Samples were mounted on aluminum SEM stubs using conductive carbon tabs (Ted Pella) and grounded using conductive silver paint (PELCO, Ted Pella). All images were captured at an accelerating voltage of 5 kV using a working distance of 5 mm and a 30 μm aperture. Both the In-lens and SE2 detectors were used for imaging.

### Microscopy (TEM)

Bright-field transmission electron microscopy (TEM) was performed using a JEOL 2100 system operating at an accelerating voltage of 200 kV. Samples were diluted and drop-cast onto holey carbon grids (Agar Scientific) for imaging. The grids were placed on filter membranes to wick away excess solvent and dried overnight at 120 °C in a vacuum oven.

### Electrical Characterization

Direct current (DC) electrical characterization of the printed networks was performed in ambient conditions using a Keithley 2612A source-meter connected to a probe station. Four-terminal measurements were used to determine the resistance of the printed AgNW networks. The thickness of the deposited traces was determined using SEM cross sections.

### Temperature-Dependent Impedance

The low frequency real component of the sample impedance was measured from a frequency of 10 to 1000 Hz in the temperature range 20 to −120 °C using a broadband α High-Resolution impedance Analyzer (Novocontrol GmbH, Germany), which utilizes a capacitance bridge technique to calculate impedance. The low frequency real impedance is expected to be identical to the DC resistance in samples such as these. The samples were placed inside a sample holder which has a fitted Pt 100 Ω resistance temperature sensor in contact with the electrodes. The temperature of the sample was controlled inside a double wall cryostat and maintained by a heated N_2_ jet produced by evaporating liquid nitrogen inside a 50 L dewar (Apollo 50 by Messer Griesheim GmbH). The Quatro temperature controller controls the power supplied to the dewar and gas heater. The AC measuring voltage applied to the sample was set at 0.5 V.

### FIB-SEM Nanotomography

FIB-SEM nanotomography (FIB-SEM NT) was performed using a ZEISS Auriga dual-beam system and ATLAS 5 software (Version 5.3.3.31, ZEISS) as described in ref (46). All images were captured at a working distance of 5 mm using a 30 μm aperture and 2 kV accelerating voltage. Printed AgNW networks on glass microscope slides were directly mounted on aluminum stubs using conductive carbon tabs (Ted Pella Inc.) and grounded using silver paint (Ted Pella Inc.). Network cross sections were milled using a 30 kV:600 pA gallium ion beam and imaged using both the In-lens and SE2 detectors. A slice thickness of 15 nm and in-plane pixel size of 5 nm were used to produce voxels 375 nm^3^ in size. The image stacks generated by the FIB-SEM NT run were aligned and interpolated using the sum of squared differences (SSD) matching algorithm in Dragonfly (Version 2022.2.0.1409, Object Research Systems). To enable quantitative analysis, the greyscale network cross sections (SE2 detector) were segmented into their nanowire and pore components using the trainable WEKA segmentation plugin^68^ in FIJI.^69^ Prior to this the pixel intensity in each stack was normalized and the brightness and contrast were adjusted globally to maximize the difference between pore and nanowire pixels in FIJI. The two resulting image stacks contained only pore or AgNW pixels, which were then converted to 3D images representing both phases using Dragonfly. Network porosity was determined from the 3D volumes using both Taufactor^70^ and FIJI.^69^

## Supporting Information Available

Table comparing literature values for the Bloch–Grüneisen fitting parameters (XLSX)
Silver nanowire size analysis; discussion on the problems with the impedance spectroscopy method (PDF)
