# Supplementary material for: Extracting the Temperature Dependence of Both Nanowire Resistivity and Junction Resistance from Electrical Measurements on Printed Silver Nanowire Networks
Source: ACS Appl Electron Mater. 2025 Jan 9;7(2):806–15. doi: 10.1021/acsaelm.4c01965 (PMC11780743; doi:10.1021/acsaelm.4c01965)
Supplement: Supplementary file 2 — el4c01965_si_002.pdf [file el4c01965_si_002.pdf]

Supporting Information for

Extracting the temperature dependence of both nanowire resistivity and junction resistance from electrical measurements on printed silver nanowire networks

Emmet Coleman,[1] Adam Kelly,[1] Cian Gabbett,[1] Luke Doolan,[1] Shixin Liu,[1] Neelam Yadav,[2] Jagdish K. Vij,[2] and Jonathan N. Coleman[1]*

[1]*School of Physics, CRANN & AMBER Research Centres, Trinity College Dublin, Dublin 2, Ireland*

[2]*Department of Electronic & Electrical Engineering, Trinity College Dublin, Dublin 2, Ireland*

*colemaj@tcd.ie (Jonathan N. Coleman); Tel: +353 (0) 1 8963859.

Contents

S1: Silver Nanowire Size Analysis S3
S2: Problems with the Impedance Spectroscopy Method.. S6
S3: Literature Bloch-Grüneisen Fitting Parameters Comparison S7
References.. S8

S1: Silver Nanowire Size Analysis

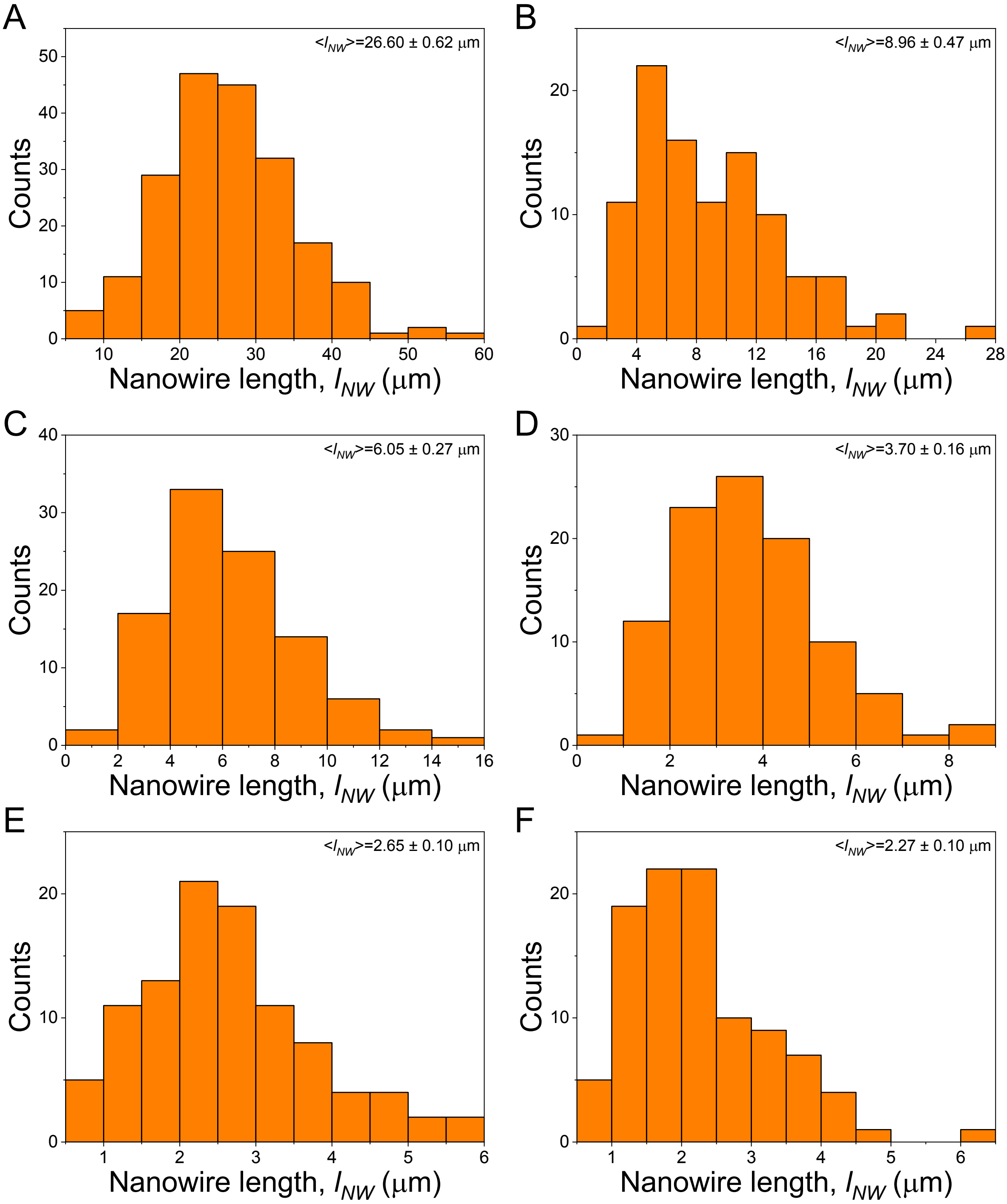

Figure S1: (A-F) SEM histograms of l_{NW} for AgNWs with a 38nm diameter having increasing sonication times of **(A)** 0, **(B)** 0.25, **(C)** 0.5, **(D)** 1, **(E)** 1.5 and **(F)** 2 hours.

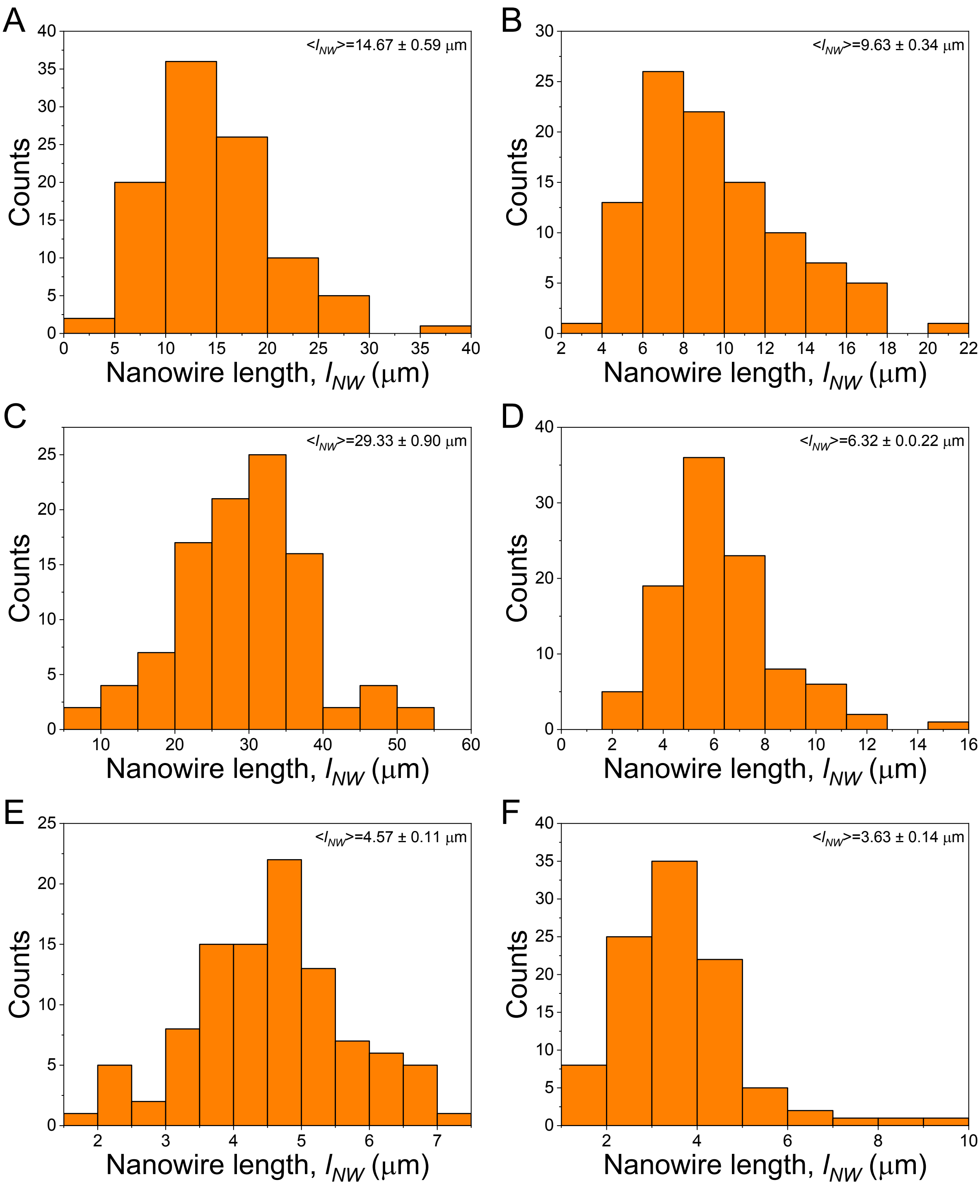

Figure S2: (A-F) SEM histograms of l_{NW} for AgNWs with a 57nm diameter having increasing sonication times of **(A)** 0, **(B)** 0.25, **(C)** 0.5, **(D)** 1, **(E)** 1.5 and **(F)** 2 hours.

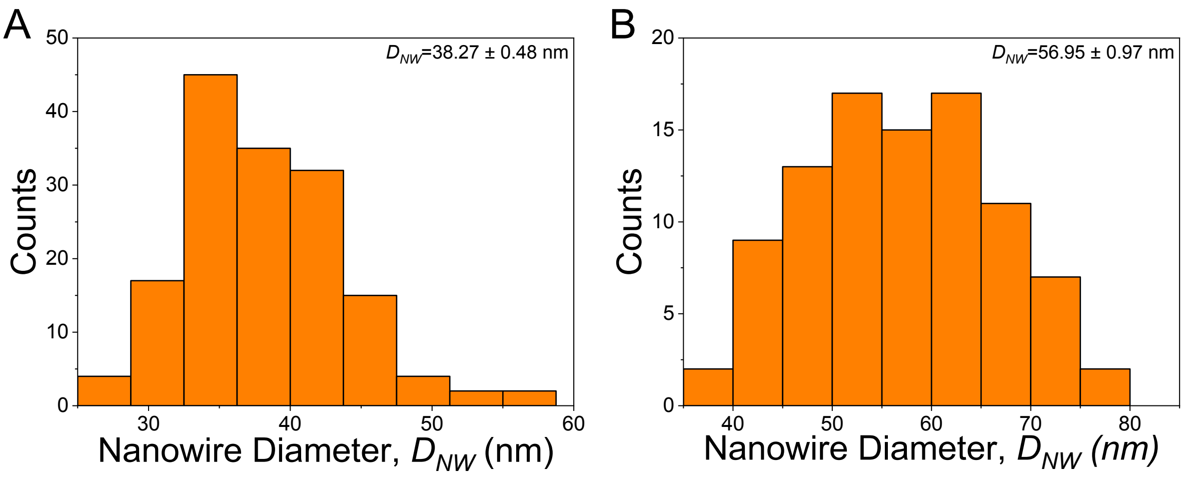

Figure S3: (A-F) SEM histograms of D_{NW} for AgNWs labelled **(A)** 38nm and **(B)** 57nm throughout this study.

S2: Problems with the Impedance Spectroscopy Method

It is not yet clear how broadly applicable the impedance spectroscopy technique described in ref[1] is to measure R_J, C_J and R_{NS}, and what other materials it can be extended to. This procedure models the combination of a nanoparticle and junction as a so-called Randles circuit: a resistor, R_N, representing the nanoparticle, in series with a parallel resistor/capacitor pair representing the junction resistance, R_J, and capacitance, C_J. The presence of the capacitor causes features to appear at high frequency (typically >10^6 rad/s) in the impedance spectra which can be fitted using equivalent circuit models to give R_N, R_J, and C_J. However, in practice such features can only be fitted when $(R_JC_J)^{-1} < \omega_{Max}$ where ω_{Max} is the highest angular frequency accessible to the impedance spectrometer. In ref 1 MoS_2 networks displayed $(R_JC_J)^{-1}$ values of ~10^7 rad/s, making them accessible to good impedance spectrometers. However, networks of conducting nanoparticles will have much lower junction resistances. In all likelihood this will increase $(R_JC_J)^{-1}$ outside the accessible range of most impedance spectrometers. Thus, an alternative method is required to simultaneously interrogate both intra- and inter-particle transport mechanisms in nanonets.

S3: Literature Bloch-Grüneisen Fitting Parameters Comparison

**Reference**	D_{NW} **(nm)**	**Network/ Single Nanowire**	Θ_D **(K)**	ρ_0 **(10^{-9} Ω m)**	$\alpha_{e\text{-}ph}$ **(10^{-8} Ω m)**
Bulk Silver			235	0.01	5.24
Zhao[2]	84	Nanowire	128	1.67	3.47
Zhao[2]	38	Nanowire	173	6.0	5.02
Cheng[3]	227	Nanowire	151	3.25	9.91
Kojda[4]	150	Nanowire	215	2.04	6.06
He 2018[5]	210	Nanowire	199	162	10.8
This Work	38	Network	181	8.9	9.6
This Work	57	Network	133	4.8	3.0

Table S1: Table comparing fitting parameters for the Bloch-Grüneisen equation for various sources found in literature.

References

(1) Gabbett, C.; Kelly, A.; Coleman, E.; Doolan, L.; Carey, T.; Synnatschke, K.; Liu, S.; Dawson, A.; O'Suilleabhain, D.; Munuera, J.; et al. Understanding how junction resistances impact the conduction mechanism in nano-networks. *NATURE COMMUNICATIONS* **2024**, *15*, Article. DOI: 10.1038/s41467-024-48614-5.
(2) Zhao, Y.; Fitzgerald, M. L.; Tao, Y.; Pan, Z. L.; Sauti, G.; Xu, D. Y.; Xu, Y. Q.; Li, D. Y. Electrical and Thermal Transport through Silver Nanowires and Their Contacts: Effects of Elastic Stiffening. *Nano Letters* **2020**, *20* (10), 7389-7396, Article. DOI: 10.1021/acs.nanolett.0c02014.
(3) Cheng, Z.; Liu, L. J.; Xu, S.; Lu, M.; Wang, X. W. Temperature Dependence of Electrical and Thermal Conduction in Single Silver Nanowire. *Scientific Reports* **2015**, *5*, 12. DOI: 10.1038/srep10718.
(4) Kojda, D.; Mitdank, R.; Handwerg, M.; Mogilatenko, A.; Albrecht, M.; Wang, Z.; Ruhhammer, J.; Kroener, M.; Woias, P.; Fischer, S. F. Temperature-dependent thermoelectric properties of individual silver nanowires. *Physical Review B* **2015**, *91* (2), 13, Article. DOI: 10.1103/PhysRevB.91.024302.
(5) He, G. C.; Lu, H.; Dong, X. Z.; Zhang, Y. L.; Liu, J.; Xie, C. Q.; Zhao, Z. S. Electrical and thermal properties of silver nanowire fabricated on a flexible substrate by two- beam laser direct writing for designing a thermometer. *Rsc Advances* **2018**, *8* (44), 24893-24899. DOI: 10.1039/c8ra03280g.
